# Preoptic Area Activation and Vasotocin Involvement in the Reproductive Behavior of a Weakly Pulse-Type Electric Fish, *Brachyhypopomus gauderio*

**DOI:** 10.3389/fnint.2019.00037

**Published:** 2019-08-13

**Authors:** Paula Pouso, Álvaro Cabana, James L. Goodson, Ana Silva

**Affiliations:** ^1^Departamento de Histología y Embriología, Facultad de Medicina, Universidad de la República, Montevideo, Uruguay; ^2^Unidad Bases Neurales de la Conducta, Departamento de Neurofisiología Celular y Molecular, IIBCE, Montevideo, Uruguay; ^3^Centro de Investigación Básica en Psicología (CIBPsi) and Instituto de Fundamentos y Métodos, Facultad de Psicología, Universidad de la República, Montevideo, Uruguay; ^4^Department of Biology, Indiana University, Bloomington, IN, United States; ^5^Laboratorio de Neurociencias, Facultad de Ciencias, Universidad de la República, Montevideo, Uruguay

**Keywords:** preoptic area, electric fish, FOS, vasotocin, social behavior

## Abstract

Social behavior exhibits a wide diversity among vertebrates though it is controlled by a conserved neural network, the social behavior network (SBN). The activity of the SBN is shaped by hypothalamic nonapeptides of the vasopressin-oxytocin family. The weakly electric fish *Brachyhypopomus gauderio* emits social electrical signals during courtship. Three types of vasotocin (AVT) cells occur in the preoptic area (POA), one of the SBN nodes. In this study, we aimed to test if POA neurons of the nucleus preopticus ventricularis anterior (PPa) and posterior (PPp), and in particular AVT+ cells, were activated by social stimuli using a 2-day behavioral protocol. During the first night, male-female dyads were recorded to identify courting males. During the second night, these males were divided in two experimental conditions: isolated and social (male with a female). Both AVT cells and the cellular activation of the POA neurons (measured by FOS) were identified. We found that the PPa of social males showed more FOS+ cells than the PPa of isolated males, and that the PPa had more AVT+ cells in social males than in isolated males. The double-immunolabeling for AVT and FOS indicated the activation of AVT+ neurons. No significant differences in the activation of AVT+ cells were found between conditions, but a clear association was observed between the number of AVT+ cells and certain behavioral traits. In addition, a different activation of AVT+ cell-types was observed for social vs. isolated males. We conclude that the POA of *B. gauderio* exhibits changes induced by social stimuli in reproductive context, involving an increase in AVT production and a different profile activation among AVT+ cell populations.

## Introduction

Social behavior in vertebrates arises as an emergent property of the social behavior network (SBN), a network of brain nuclei that includes the medial preoptic area (POA), lateral septum, anterior hypothalamus, ventromedial hypothalamus, periaqueductal gray, medial amygdala, and bed nucleus of the stria terminalis (Newman, 1999; Nelson and Trainor, 2007). It is accepted that the diversity in social behavior, within a given species and across vertebrates, would be achieved by changes in the distributed pattern of neural activity among the interconnected nodes of the SBN (Newman, 1999; Goodson and Kabelik, 2009). These neural circuits, initially described in mammals, appear to be highly conserved among all classes of vertebrates (Goodson, 2005; O’Connell and Hofmann, 2011). Multiple neuromodulators shape the spatio-temporal pattern of activity of the SBN controlling the emergence of environmental, ontogenic, social context, and phenotype-dependent behaviors (Newman, 1999; Goodson et al., 2012; Johnson and Young, 2017).

One effective way to approach the neural circuits underlying complex social behavior is the use of immediate early genes (IEGs) as neural activity markers (Clayton, 2000; Kovács, 2008). Across vertebrates, several studies report an increased expression of IEGs in specific nodes of the SBN in association to courtship (Kollack-Walker and Newman, 1995; Curtis and Wang, 2003; Okuyama et al., 2011), parental care (O’Connell et al., 2012; Zhong et al., 2014; Kasper et al., 2018; Kent and Bell, 2018), territorial behavior (Kollack-Walker and Newman, 1995; Goodson and Evans, 2004; Teles et al., 2015) and grouping (Goodson et al., 2005a; Cabrera-Álvarez et al., 2017; Wilson et al., 2018). The use of IEGs as a proxy for visualizing the activation of brain areas associated with social behavior has also implicated the activation of hypothalamic neurons involved in the production of nonapeptides of the vasopressin/oxytocin family during these behaviors (Goodson and Wang, 2006; Goodson and Kabelik, 2009; O’Connell et al., 2012; Loveland and Fernald, 2017; Wilson et al., 2018). Only two previous studies in teleosts have found a differential activation between isolated and social animals of isotocin (oxytocin homolog) neurons associated with parental care (O’Connell et al., 2012) and of vasotocin (AVT, vasopressin homolog) neurons involved in courtship and aggression (Loveland and Fernald, 2017). However, these studies failed to find the general pattern of increased expression of IEGs in social animals in the brain areas in which these nonapeptidergic neurons occur.

Weakly electric fish are traditional neuroethological model systems that exhibit both locomotor and electric displays in their behavior. These fish emit an electric organ discharge (EOD) generated by a very well-known electromotor circuit. The EOD rate and waveform contain information about an individual’s species identity, sex, and physiological state (Stoddard, 2002; Caputi et al., 2005). *Brachyhypopomus* *gauderio* (Giora and Malabarba, 2009), former *Brachyhypopomus pinnicaudatus* (Hopkins, 1991) is a south American freshwater weakly electric fish that belongs to the Order Gymnotiformes. This species is gregarious, has a polygynous breeding system, and exhibits during the breeding season a strong morphological and electrophysiological sexual dimorphism (Caputi et al., 1998; Silva et al., 2003).

The behavioral displays of both courting (male-female) and agonistic (male-male) dyadic interactions are well understood in this species (Perrone et al., 2009; Zubizarreta et al., 2012). It is also clear that the electrical signaling of social behavior in this species is modulated by AVT in a context-dependent manner. For example, the nocturnal increase in EOD rate is AVT-dependent in courting breeding pairs (Silva et al., 2007; Perrone et al., 2010) but not in isolated individuals (Migliaro and Silva, 2016). In addition, males *B. gauderio* signal dominance by an AVT-dependent increase in EOD rate that is not observed in subordinates (Perrone and Silva, 2016). Further, as in other teleosts (Batten et al., 1990; Holmqvist and Ekström, 1995; Goodson and Kabelik, 2009; Ramallo et al., 2012), the typical three types of AVT+ cells [parvocells (pPOA), magnocells (mPOA), and gigantocells (gPOA)], have been exclusively reported within the POA of this species projecting to several brain areas including those related to the control of electromotor behavior (Pouso et al., 2017). In weakly electric fish, AVT+ cells are organized in two nuclei within the POA described by Maler et al. (1991): the preopticus periventricularis anterior (PPa) and the preopticus periventricularis posterior (PPp).

In this study, we aimed to evaluate how social stimulation (the presence of the female) affected the activation of POA neurons in general, and of AVT cells in particular, in courting males of *B. gauderio*. For the first time in electric fish, we double-immunolabeled POA neurons with AVT and FOS and showed that: (a) the PPa of chirping males shows increased transcription for FOS after the social stimulus of the female; (b) the number of AVT cells is higher in social males with respect to isolated ones in the PPa; and (c) the activation profile of AVT cells types is different between social and isolated males.

## Materials and Methods

### Animals

In this study, we used 24 breeding adult males of *Brachyhypopomus gauderio* (Giora and Malabarba, 2009), with body-length ranging from 14.5 to 19.3 cm and body-weight from 5.2 to 8.9 g. Fish were detected and collected during the breeding season (November-February) in a freshwater lagoon in Laguna Lavalle (31°48′S, 55°13′W, Department of Tacuarembó, Uruguay) using a “fish detector,” an electronic audio amplifier connected to a pair of electrodes, as previously described (Silva et al., 2003).

Fish were housed for at least 10 days before the behavioral experiments in 500-L outdoor communal tanks with two males and six females, which replicate the sex ratio of a natural breeding population (Miranda et al., 2008). All environmental variables were kept within the normal range observed in the natural breeding habitat (Silva et al., 2003). Water conductivity was maintained below 200 μS/cm by the addition of deionized water. Water temperature in the tanks ranged from 18 to 33°C and the natural photoperiod ranged from LD14:10 to LD13:11. The surface of the water was covered with aquatic plants (*Eichhornia crassipes, Pistia stratiotes, Salvinia sp*.) that provided shelter for the fish. Fish were fed *Tubifex tubifex*. The fish collection, transportation, housing, and recording conditions were adjusted in order to minimize stress and to achieve reliable and repeatable behaviors. All experiments were performed in accordance with institutional and national guidelines and regulations for animal welfare. This study was reviewed and approved by an ethics committee (Comisión Honoraria de Experimentación Animal, Universidad de la República, Protocol Number 008/002).

### Behavior

#### Behavioral Recording Station

Fish simultaneous video and electric recordings were performed in an experimental setup previously described (Silva et al., 2007). Four experimental tanks (50-l glass aquaria, 55 × 40 × 25 cm) were fitted with two pairs of orthogonal electrodes attached to each tank wall. The physicochemical parameters (water temperature, conductivity, and pH) and the day–night cycle of indoor tanks matched those of the outdoor housing tanks. All the experiments were performed in total darkness illuminated by an array of infrared LEDs (L-53F3BT) located above the tank and an infrared-sensitive video camera (SONY CCD-Iris and RoHS CCD Digital Video Camera) focused on the bottom of the tank. The detection of electric signals of freely moving fish was done by two pairs of fixed electrodes, connected to two high-input impedance amplifiers (FLA-01, Cygnus Technologies Inc.). Images and electric signals were captured by a video card (Pinnacle Systems, PCTV HD pro stick) and stored in the computer for further analysis. Fish were placed for 6 h in the recording tank at constant temperature (27–29°C) before the beginning of the behavioral experiments.

#### Behavioral Paradigm

**Figure 1:**
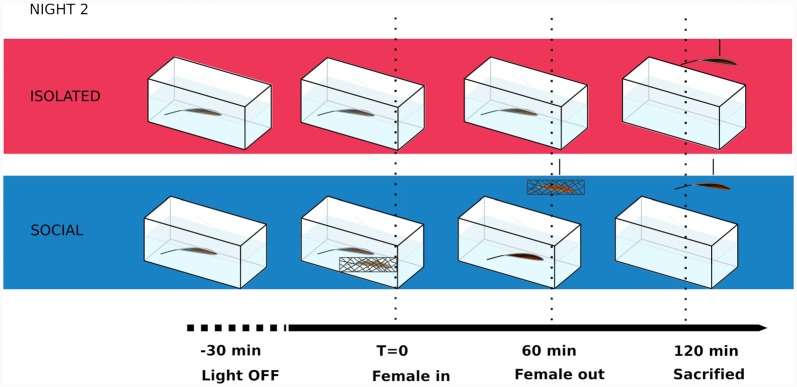
Schematic of behavioral paradigm during the second night in courting males of *Brachyhypopomus gauderio*. T, time.

All males used in this study were tested in a 2-day protocol. The courting behavior of male-female dyads (originally housed in the same tank) was tested during the first night. To proceed to the second night of the experiment, we selected the dyads that displayed locomotor and electric courting behavior as previously described in this species (Perrone et al., 2009). In other words, we only selected the dyads in which males chirped and females turned off their electric discharges. Both males and females were isolated for more than 24 h in individual tanks after the first night experiment. In the second night (Figure 1), the chirping males were randomly assigned to two experimental conditions: social (*n* = 8) and isolated (*n* = 6). Isolated males were recorded 150 min after artificial sunset. Social males were also recorded 150 min after artificial sunset, first in isolation until the same female used in the first night (immobilized in a net) was added to the tank 30 min after artificial sunset and removed 60 min later (Figure 1). Both isolated and social males were simultaneously anesthetized 120 min after the female was removed, intracardiac-perfused and brains were dissected for immunohistochemistry. In another control experiment, we carried out the same 2-day protocol to test, in the second night, isolated males vs. isolated males plus the addition of the empty net.

#### Behavioral Data Processing

We measured the following behavioral parameters in courting males in all the experiments: the time of movement of animals (measured in percentage), the number of approaches towards female, total time with female and the number of chirps emitted. Data for one male-female dyad was lost due to corruption of the output video file. Hence, behavior analyses are only shown for only seven animals.

### Tissue Collection and Processing

#### Double Immunolabeling for FOS and AVT

Fish were anesthetized by immersion in 0.05% 2-phenoxy-ethanol (Sigma, P-1126) and then perfused with saline followed by 4% paraformaldehyde in phosphate buffered saline (PBS, 25–35 ml; pH = 7.4). Coded brains were processed by an observer blinded to treatment. Brains were dissected, post-fixed overnight in the same fixative at 4°C, rinsed in 0.1 M PBS and cryoprotected in 30% sucrose for 24 h at 4°C before embedding in Cryomatrix (Thermo Scientific) and storage at −80°C until processing. Brains were then sectioned on a cryostat into three series of 50 μm sections. The first series was double immunolabeling for FOS and AVT. Free-floating sections were rinsed in PBS, and non-specific binding sites were blocked with normal 10% donkey serum (DS, Jackson Immunoresearch) + 0.3% Triton (Sigma) in PBS 0, 1 M; PH 7, 4 for 1 h. Sections were incubated for 36 h in primary antibodies (anti- FOS, 1:500, goat, custom from Bethyl Labs) ; anti-AVP, 1:500, rabbit, Immunostar, dissolved in 0.1 M PBS + 0.3% Triton X-100 + 5% DS with sodium azide 0.01%. After incubation with the primary antibodies, sections were rinsed (3 × 10 min in PBS) and incubated for 2 h at room temperature with secondary antibodies [Alexa Fluor 594 (red) donkey anti-goat IgG (H + L), 1:200, Invitrogen, Cat#A11058, and Alexa Fluor 680 (false color green) donkey anti-rabbit IgG (H + L), 1:200, Invitrogen, Cat#A21109] dissolved in 0.1 M PBS + 0.3% Triton X-100 + 5% DS with Sodium Azide 0.01%. All sections were then rinsed (3 × 10 min in PBS) and mounted with a nuclear stain (ProLong Gold Antifade Mountant with DAPI, Molecular Probes, ‘Cat#P3693).

**Figure 2:**
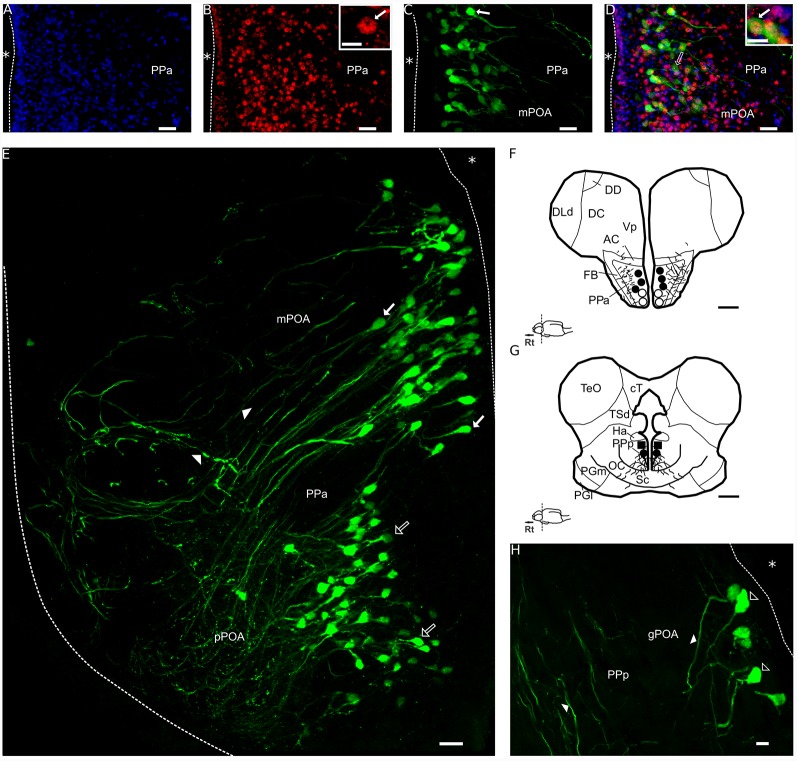
Double immunolabeling for FOS and AVT in a transverse section of the POA of a social male of *Brachyhypopomus gauderio*. **(A)** Immunolabeling for DAPI (blue). **(B)** Immunolabeling for FOS (red). Inset in **(B)**: the arrow shows the nuclear label for FOS. **(C)** Immunolabeling for AVT (green). The arrow indicates an AVT+ cell. **(D)** Merge of **(A–C)**. Blank arrow indicates an AVT+ cell. Inset in **(D)**: the arrow shows a double-immunolabeled cell for FOS and AVT (yellow). **(E)** PPa section of the POA immunolabeled for AVT (false color green), blank arrows indicate pPOA cells and filled arrows indicate mPOA cells. Arrowheads indicate AVT+ fibers. **(F,G)** Diagrams of AVT+ cells; pPOA (dots), mPOA (black dots), gPOA (squares) and fibers (lines) in a rostral **(F)** and caudal **(G)** transverse sections. **(H)** PPp section of the POA immunolabeled for AVT (false color green). Blank arrowheads indicate gPOA cells somata and filled arrowheads indicate fibers. *, ventricle all levels; Rt, rostral; AVT, vasotocin; pPOA, parvocells; mPOA, magnocells; gPOA, gigantocells; PPa, nucleus preopticus ventricularis, anterior subdivision; PPp, nucleus preopticus ventricularis, posterior subdivision; AC, anterior commissure; cT, tectal commissure; DC, central division of dorsal forebrain; DD, dorsal division of the dorsal forebrain; Dld, dorsolateral telencephalon, dorsal subdivision; FB, forebrain bundle; Ha, hypothalamus anterioris; OC, optic chiasm; PGl, preglomeriular nucleus, lateral subdivison; PGm, preglomerular nucleus, medial subdivison; nucleus; PPa, nucleus preopticus periventricularis, anterior subdivision; Sc, suprachiasmatic nucleus; TeO, optic tectum; TSd, torus semicircularis, dorsal subdivision; Vp, ventral telencephalon, posterior subdivision. Bar scale: **(A–D)**: 20 μm; **(E)**: 20 μm; **(F,G)**: 500 μm; **(H)**: 10 μm. Inset in **(B)**: 5 μm. Inset in **(D)**: 10 μm.

AVT+ cells are only present in the POA forming a large band extended from behind the anterior commissure to the posterior POA, above the optic chiasm (Pouso et al., 2017, Figures 2F,G). Maler et al. (1991) identified two nuclei within the POA in which AVT+ cells were later reported to occur: the PPa (Figures 2A–F) and the PPp (Figures 2G,H). Parvocellular AVT cells (pPOA) were exclusively identified in the PPa, gigantocellular AVT cells (gPOA) were exclusively in the PPp while magnocellular (mPOA) cells occur in both PPa and PPp (Pouso et al., 2017).

Double-immunolabeling for FOS and AVT was performed and quantified in PPa and PPp sections of the POA in both experimental conditions and an example is shown in a PPa section of a social male (Figures 2A–D). Control sections were incubated with the primary antiserum (anti-AVP) pre-absorbed with an excess of AVT (1 μg/ml; Cat. 66-0-09, American Peptide Company Inc., Sunnyvale, CA, USA) and no labeling was present. On the other hand, the preabsorption of anti AVP with an excess of IST (10 μM, 1:500, Bachem) showed a labeling that did not differ with plain AVP antibody staining (data not shown). Control sections were incubated with the primary antiserum (anti-FOS) pre-absorbed with an excess of FOS (1 mg/ml) for 3 h at room temperature and no labeling was observed (data not shown). Control experiments omitting the primary and secondary antibody were routinely performed.

### Image Acquisition and Cell Counting

Images were generated at 10× using a Zeiss AxioImager microscope outfitted with an AxioCam HRm, z-drive, and an Apotome optical dissector (Carl Zeiss Inc.). We used standardized methods to quantify FOS+ cells (Goodson et al., 2005b; O’Connell et al., 2012; Lin et al., 2018). To measure the density of FOS+ cells, the counts of FOS+ nuclei were conducted within standardized polygons or boxes (100 μm^2^) that were superimposed on the digital photomicrographs using Gimp 2.8.16 software. Dots were placed over each labeled cell (in a separate Gimp layer) and the dots were then counted using ImageJ software (National Institutes of Health, Bethesda, MD, USA). The raw cell counts were ultimately converted into the number of FOS+ nuclei per 100 μm^2^ of tissue.

We also quantified the total number of AVT+ cells per slice following Pouso et al. (2017). Only somata with a distinct perimeter and at least one neurite were measured. Given that AVT immunostaining was conducted on 50 μm thickness slices and that soma size of these cells is ~12–20 μm in diameter, it is unlikely to count them twice. Further, we avoided this possibility by not using adjacent slices. To quantify double labeling cells for FOS and AVT we use monochrome photomicrographs for each fluorophore at 10 and then quantification was subsequently conducted from layered monochrome images using Gimp 2.8.16 and ImageJ software (National Institutes of Health, Bethesda, MD, USA). We also quantify the proportion of these neurons co-labeled with FOS using methods previously described (Goodson and Evans, 2004; Goodson and Wang, 2006).

### Statistical Analysis

Immunohistochemical quantification data were analyzed using general and generalized linear mixed models (Faraway, 2016). These models (also known as nested, hierarchical or multilevel models) explicitly consider the existence of correlations of observational units coming from the same individual, extend the applicability of linear models beyond the case of normally distributed outcomes, and are being increasingly used in ecology and neuroscience (for instance see Boisgontier and Cheval, 2016; Harrison et al., 2018; Fischer et al., 2019). Experimental condition (isolated, social), anatomical or morphological descriptors such as POA section (PPa or PPp) or cell type (gPOA, mPOA or pPOA), and behavioral predictors were introduced as fixed effects, while individual was included as random intercept effects. In some cases, the significance of main effects and interactions was assessed through analysis of variance (or deviance). *Post hoc* pairwise comparison of *p*-values were adjusted using the multivariate-*t*-test (mvt) method. Cell counts (AVT+ or FOS+ cells) for each slice were analyzed using generalized linear mixed models with Poisson distributions using the logarithmic link. Proportions (AVT+ cells that are also FOS+) for each slice were analyzed using generalized linear mixed models with binomial distributions using the logit link. The relationship between total movement and experimental condition was explored using a general linear model with experimental condition as a fixed effect. All analyses were programmed in the R statistical programming language.

## Results

The double immunolabeling for AVT and FOS was performed in PPa and PPp sections of the POA in social and isolated males. An example of a PPa section is shown in a social male in Figures 2A–D. The nuclei of cells were stained with DAPI (Figure 2A), which allows the clear identification of double immunostained cells (Figure 2D, inset). The nuclear labeling for FOS is shown in the PPa (Figure 2B); the labeling is distributed as a “ring” inside the nucleus of the cell (inset in Figure 2B). AVT labeling was present in somata and fibers of cells in the PPa (Figure 2C) as well as the double labeling for FOS and AVT (Figure 2D). In accordance to the previous description of the POA of this species (Pouso et al., 2017), the distribution of AVT pPOA and mPOA cells is more rostral and ventral while gPOA cells are only located in the most caudal and dorsal portions of the POA (Figures 2E–H).

**Figure 3:**
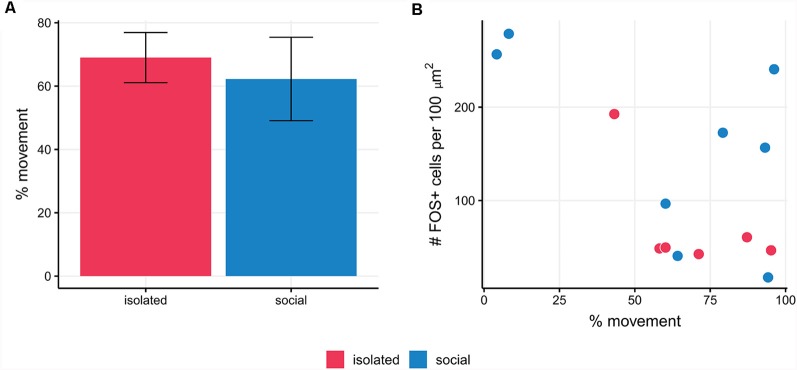
**(A)**The percentage of movement in social males and isolated ones was similar (*p* = 0.69). **(B)** The mean number of FOS+ cells per slice is not correlated with the percentage of movement in social and isolated males of *B. gauderio* (*p* = 0.18).

We assessed whether locomotor activity affected FOS labeling (Montag-Sallaz et al., 1999) and we quantified the percentage of time in which social and isolated males displayed locomotor activity. We found no correlation between the number of FOS+ immunolabeled cells and the percentage of time in which social and isolated males display locomotor activity [Generalized linear mixed model (Poisson): Wald’s $\chi_{\left(1\right)}^{2}$ = 2.03, *p* = 0.15]. Moreover, the percentage of movement in social males and isolated ones was similar (isolated: 69%; social: 62.25%; *t*_(12)_ = −0.4; *p* = 0.69; Figure 3A). We also validated the evaluation of the presence of the female (inside the net) as the actual social stimulus by checking that the net itself was not a relevant stimulus for FOS expression in isolated courting males [mean number of FOS+ cells per 100 μm^2^ in isolated males, vs. isolated males with empty net; Generalized linear mixed model (Poisson): Wald’s $\chi_{\left(1\right)}^{2}$ = 1.79; *p* = 0.18].

### Social Stimuli Increase FOS Expression in the Preoptic Area of *B. gauderio* Courting Males

**Figure 4:**
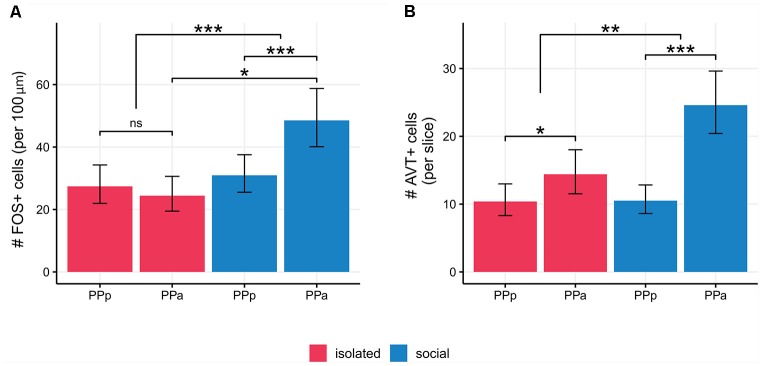
**(A)** Social males show higher number of FOS+ cells/100 μm^2^ in PPa sections with respect to PPp ones (*p* < 0.001) and as compared to PPa sections in isolated males (*p* = 0.02). **(B)** Isolated males show a higher number of AVT+ cells in PPa sections compared to PPp sections. (*p* = 0.01). Social males also show a higher number of AVT+ cells in PPa sections compared to PPp section (*p* < 0.001). The difference between PPa and PPp cell AVT+ cells was larger for social males (*p* = 0.003). POA, preoptic area; PPa, nucleus preopticus ventricularis, anterior subdivision; PPp, nucleus preopticus ventricularis, posterior subdivision. *p < 0.05, **p < 0.01, ***p < 0.001; ns, non-significant.

We quantified the number of FOS+ cells per 100 μm^2^ in PPa and PPp sections of the POA in social and isolated males (Figure 4A, Table 1). The density of FOS+ cells in PPa and PPp is different between experimental conditions [Generalized linear mixed model (Poisson). Interaction term: condition × POA section: Wald’s $\chi_{\left(1\right)}^{2}$ = 23.99; *p* = 9.7 × 10^−7^; Figure 4A, Table 1]. In the POA of isolated males, no differences were found between the mean number of FOS+ cells in PPa and PPp sections (paired contrast: *z* = 1.23, *p* = 0.21, Figure 4A, Table 1). In contrast, within the POA of social males, the mean number of FOS+ cells is higher in PPa sections compared to PPp ones (*z* = −6.68; *p* = 2.37 × 10^−11^, Figure 4A, Table 1). Furthermore, within the PPa sections of the POA, the mean number of FOS+ cells was higher in social males as compared to isolated males (*z* = 2.32; *p* = 0.02, Figure 4A, Table 1).

**Table 1:** Summary of main results for FOS+, AVT+ and FOS+/AVT+ cells quantification in POA sections per experimental conditions.

Exp. condition	POA section or POA cell type	Mean (per slice)	95%CI
#FOS+ cells			
isolated	PPp	27.42	(17.71–42.43)
isolated	PPa	24.42	(15.68–38.04)
social	PPp	30.95	(21.2–45.18)
social	PPa	48.54	(33.39–70.57)
#AVT+ cells			
isolated	PPp	10.39	(6.71–16.09)
isolated	PPa	14.41	(9.3–22.33)
social	PPp	10.52	(7.12–15.54)
social	PPa	24.6	(17.09–35.42)
Proportion of FOS+/AVT+ cells			
isolated	gPOA	0.34	(0.11–0.67)
isolated	mPOA	0.33	(0.17–0.54)
isolated	pPOA	0.1	(0.03–0.34)
social	gPOA	0.63	(0.27–0.89)
social	mPOA	0.18	(0.1–0.31)
social	pPOA	0.07	(0.02–0.2)

*POA, preoptic area; PPa, nucleus preopticus ventricularis, anterior subdivision; PPp, nucleus preopticus ventricularis, posterior subdivision.

### Social Males Exhibit Changes in the Number of AVT+ Cells

We quantified the number of AVT+ cells per slice in PPa and PPp sections of the POA both in social and isolated males (Figure 4B, Table 1). In both experimental conditions, we found a higher number of AVT+ cells per slice in the POA in PPa sections, as compared to the PPp (isolated: *z* = 2.53, *p* = 0.01; social: *z* = 7.07 *p* = 1.52 × 10^−12^, Figure 4B, Table 1). However, the difference between PPa and PPp cell counts was larger for social males than for isolated males [Generalized linear mixed model (Poisson). Interaction term: condition × POA section: Wald’s $\chi_{\left(1\right)}^{2}$ = 8.78, *p* = 0.003; Supplementary Table S1]. This difference seems to be due to an increase in the number of PPa AVT+ cells in the social condition when compared to the isolated condition (*z* = 1.8, *p* = 0.06), rather than to a change in PPp cell counts between conditions (*z* = 0.04, *p* = 0.97).

### The Proportion of AVT+/FOS+ Cell Types Is Different Between Social and Isolated Males

**Figure 5:**
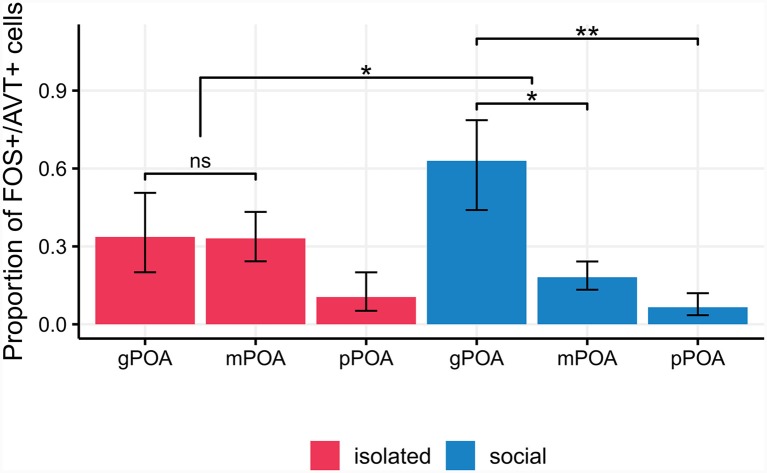
Isolated males show no significant differences in the proportion of FOS+/AVT+ cells between the different POA cell types (pairwise *p*-values > 0.14). In social males, the proportion of FOS+/AVT+ is significantly higher for gPOA cells than for mPOA cells (*p* = 0.02), and pPOA cells (*p* = 0.003). This difference in the activation profile of gPOA and mPOA cell types between experimental conditions is statistically significant (*p* = 0.03). pPOA, parvocells; mPOA, magnocells; gPOA, gigantocells. *p < 0.05, **p < 0.01, ***p < 0.001; ns, non-significant.

We found no differences in the proportion of double labeled (AVT+/FOS+) cells in the PPa and PPp of isolated and social males [Generalized linear mixed model (Binomial): Wald’s $\chi_{\left(1\right)}^{2}$ = 1.93, *p* = 0.16; Interaction term, condition × POA section: Wald’s $\chi_{\left(1\right)}^{2}$ = 0.6, *p* = 0.44; Supplementary Table S1]. Nor was the activation of each AVT+ cell type different between experimental conditions (pairwise contrasts: gPOA: *z* = −1.16, *p* = 0.24; mPOA: *z* = 1.42, *p* = 0.15; pPOA: *z* = 0.51, *p* = 0.61). However, the pattern of activation of AVT+ cell types was different between isolated and social males (Figure 5, Table 1). Isolated males showed no significant differences in the proportion of FOS+/AVT+ cells between the different POA cell types (gPOA-mPOA: *z* = 0.04, *p* = 0.99; gPOA-pPOA: *z* = 1.62, *p* = 0.39; mPOA-pPOA: *z* = 2.15, *p* = 0.14). In contrast, in social males the proportion of FOS+/AVT+ was significantly higher for gPOA cells than for mPOA cells (*z* = 2.78, *p* = 0.02), and pPOA cells (*z* = 3.41, *p* = 0.003). This difference in the activation profile of gPOA and mPOA cell types between both experimental conditions is statistically significant (interaction contrast: *z* = 2.11, *p* = 0.03).

### Social Males’ AVT Cellular Changes Correlate With Behavioral Traits

**Figure 6:**
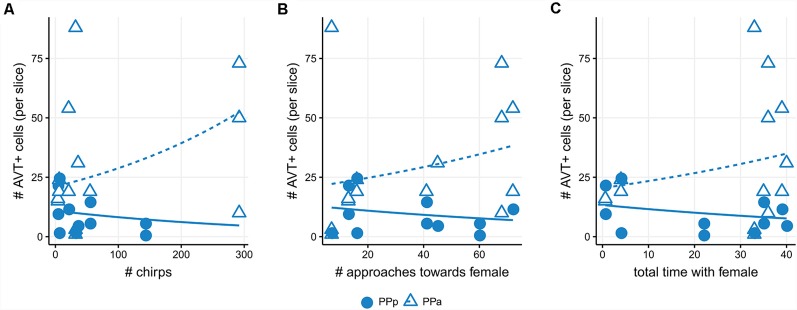
Number of AVT+ cells correlate with behavioral traits in social males. **(A)** Positive correlation between the number of AVT+ cells in PPa sections and chirp number (triangles; *p* < 0.001). Negative correlation between the number of AVT+ cells in PPp sections and chirp number (dots; *p* < 0.001). **(B)** Positive correlation between the number of AVT+ cells in PPa sections and approaches towards a female (triangles; *p* < 0.001). Negative correlation trend between the number of AVT+ cells in PPp sections and approaches towards a female (dots; *p* = 0.08). **(C)** Positive correlation between the number of AVT+ cells in PPa sections and total time with the female (triangles; *p* = 0.001). Negative correlation between the number of AVT+ cells in sections and total time with the female (dots; *p* = 0.03).

The number of AVT+ cells in PPa sections showed a positive correlation with the number of chirps displayed by social males [Generalized linear model (Poisson): *β* =0.19, Wald’s $\chi_{\left(1\right)}^{2}$ = 10.47, *p* = 0.0001, Figure 6A, Supplementary Table S1], the number of approaches towards female (*β* = 0.21 Wald’s $\chi_{\left(1\right)}^{2}$ = 12.03, *p* = 0.0005, Figure 6B, Supplementary Table S1) and the total time spent with the female (*β* = 0.29, Wald’s $\chi_{\left(1\right)}^{2}$ = 10.07, *p* = 0.001, Figure 6C, Supplementary Table S1). Meanwhile, in PPp sections of the POA, the number of AVT+ cells showed a negative correlation with chirp number (*β* = −1.0, Wald’s $\chi_{\left(1\right)}^{2}$ = 12.85, *p* = 0.0003, Figure 6A, Supplementary Table S1) and with the total time spent with the female (*β* = −0.26, Wald’s $\chi_{\left(1\right)}^{2}$ = 4.61, *p* = 0.03, Figure 6C, Supplementary Table S1), and a marginal negative correlation with the number of approaches towards female (*β* = −0.22, Wald’s $\chi_{\left(1\right)}^{2}$ = 2.99, *p* = 0.08, Figure 6B, Supplementary Table S1).

## Discussion

In this study, we confirmed for the first time in electric fish that the neuronal transcriptional FOS activity in the PPa portion of the POA, a node of the SBN, was significantly higher in courting males exposed to a social stimulus than in isolated males, as reported in other vertebrates (Kollack-Walker and Newman, 1995; Curtis and Wang, 2003; Okuyama et al., 2011). In addition, social males had more AVT+ cells in the PPa than isolated males, and the number of AVT+ cells in social males correlated with electric and locomotor courting traits. Furthermore, the profile of activation of the different AVT+ cell types differed between social and isolated males.

To confirm that the observed differences in FOS expression between experimental conditions were due only to the presence/absence of the female, the following precautions were taken. First, as FOS expression can be induced by animal movement (Montag-Sallaz et al., 1999), we confirmed that FOS expression was not correlated with the amount of movement (Figure 3). Second, it could be argued that because the experimental conditions differed in the presence or absence of both the female and the holding net, the possibility that the observed differences in FOS expression were simply due to the presence of the net could not be completely ruled out. However, in a control experiment, we found no differences in FOS expression between isolated males and males that were placed near an empty net. Hence, the net can be considered as a neutral stimulus, not inducing substantial FOS expression. Finally, although the differences found between experimental conditions could be due in part to individual differences in AVT cell counts, the inclusion of random intercepts for each animal in the regression models reduces this possibility.

We showed for the first time in an electric fish that social interaction promotes the activation of POA cells as previously described across vertebrates (Figure 4; Kollack-Walker and Newman, 1995; O’Connell et al., 2012; Wilson et al., 2018). Among teleosts, other reports showed this expected result (Teles et al., 2015; Cabrera-Álvarez et al., 2017), while others failed to find differences between social males and controls in the activation of POA cells (O’Connell et al., 2012; Loveland and Fernald, 2017). Hence, this work supports the idea of a conserved role of the POA in the modulation of social behavior in vertebrates.

Numerous studies have identified a role for both AVP and AVT in stimulating sexual, affiliative and communicative behaviors across vertebrates (Goodson and Bass, 2001; De Vries and Panzica, 2006). Particularly in teleosts, changes in the number and size of AVT cells have been reported in association with reproductive behavior (Ota et al., 1999; Grober et al., 2002; Maruska et al., 2007). In this study, we confirmed that the number of AVT cells in both social and isolated males is higher in the PPa of the POA respect to the PPp as expected from the differential rostro-caudal distribution of AVT cells in this species (Figure 2; Pouso et al., 2017). In addition, the number of PPa AVT+ cells is higher in social males than in isolated males (Figure 4B). This observation cannot be interpreted as the results of the generation of new AVT cells induced by the social stimulus, but rather as the consequence of an increase in AVT production that leads to the increase in the number of AVT cells that reach the threshold of AVT immunoreactive detection (Milo and Phillips, 2016). The presence of the female, as a sensory input, can regulate early gene expression (Ball and Balthazar, 2001) and also may activate AVT gene-related peptide release as occur with other peptides (Morton and Hutchison, 1989). Therefore, in line with the knowledge of neuropeptidergic physiology (Thompson and Walton, 2004), we interpret that the production and further liberation of AVT is involved in the reproductive behavior of *B. gauderio*.

The association of the behavioral and cellular data strongly reinforces this interpretation as we were able to find a positive correlation between the number of PPa AVT+ cells and both locomotor and electric displays of social courting males (Figure 6). On the other hand, since the number of PPp AVT+ cells is negatively correlated with chirps and locomotor traits, it is possible that cells in this portion of the POA may have a different functional role. In teleost fish, there is little information regarding the projection targets of different AVT POA cell populations. In general terms, it is known that rostral portions project to the telencephalon, thalamus and hypophysis, while caudal portions project to the telencephalon (Schreibman and Halpern, 1980; Holmqvist and Ekström, 1995; Saito et al., 2004; Dewan et al., 2008). In electric fish, anatomical and functional connections between PPa and areas that modulate the EOD have been established (Wong, 1997, 2000; Perrone et al., 2010; Pouso et al., 2017). This could indicate that different portions of the POA could project to areas that modulate different behaviors (Maruska et al., 2007), hence showing inverse correlation patterns with reproductive behavior traits.

We failed to find significant differences in activation of the different AVT cell-types between social and isolated males, probably due to the low number of slices from each animal that were processed for this experiment. However, we found differences in the activation profile of AVT cell types between both experimental conditions (Figure 5). In particular, in social males, gPOA cells turn out to be more activated with respect to mPOA and pPOA cells. Although gPOA cells are the less abundant AVT cell-type within the POA, previous reports have also emphasized the role of gPOA cells in the modulation of aggressive, reproductive and cooperative behaviors (Greenwood et al., 2008; Dewan and Tricas, 2011; Mendonça et al., 2013).

While the increased activation of the neurons in the PPa portion of the POA of courting males is very clear, this cannot be solely explained by an increased activation of AVTergic neurons. Although our results indicate that the number of AVTergic cells differs between experimental conditions and suggest that social interaction increases the activation of gPOA cells, they also suggest that other (AVT negative) cellular types of the POA are also significantly activated. These non-AVT cell populations may be more closely related to the responsiveness of the POA in a reproductive context.

## Data Availability

The datasets generated for this study are available on request to the corresponding author.

## Ethics Statement

Comisión Honoraria de Experimentación Animal, Universidad de la República, Protocol Number 008/002.

## Author Contributions

PP, JG and AS: conceptualization, investigation and resources. PP, ÁC, JG and AS: methodology. PP and AS: writing—original draft and funding acquisition. PP, ÁC and AS: writing—review and editing. JG and AS: supervision.

## Conflict of Interest Statement

The authors declare that the research was conducted in the absence of any commercial or financial relationships that could be construed as a potential conflict of interest.

## Supplementary Material

TABLE S1 Detail of the main statistical analyses reported in the text.

## References

[B1] Ball G. F. Balthazar J. (2001). Ethological concepts revisited: immediate early gene induction in response to sexual stimuli in birds. Brain Behav. Evol. 57, 252–270. 10.1159/000047244 11641562

[B2] Batten T. F. Cambre M. L. Moons L. Vandesande F. (1990). Comparative distribution of neuropeptide-immunoreactive systems in the brain of the green molly, *Poecilia latipinna*. J. Comp. Neurol. 302, 893–919. 10.1002/cne.903020416 2081820

[B3] Boisgontier M. P. Cheval B. (2016). The anova to mixed model transition. Neurosci. Biobehav. Rev. 68, 1004–1005. 10.1016/j.neubiorev.2016.05.034 27241200

[B4] Cabrera-Álvarez M. J. Swaney W. T. Reader S. M. (2017). Forebrain activation during social exposure in wild-type guppies. Physiol. Behav. 182, 107–113. 10.1016/j.physbeh.2017.10.012 29031547

[B6] Caputi A. A. Carlson B. A. Macadar O. (2005). “Electric organs and their control,” in Electroreception. Springer Handbook of Auditory Research, eds Bullock T. H. Hopkins C. D. Popper A. N. (New York, NY: Springer), 410–451.

[B5] Caputi A. A. Silva A. C. Macadar O. (1998). The electric organ discharge of *brachyhypopomus pinnicaudatus*. The effects of environmental variables on waveform generation. Brain Behav. Evol. 52, 148–158. 10.1159/000006559 9693161

[B7] Clayton D. F. (2000). The genomic action potential. Neurobiol. Learn. Mem. 74, 185–216. 10.1006/nlme.2000.3967 11031127

[B8] Curtis J. T. Wang Z. (2003). Forebrain c-*fos* expression under conditions conducive to pair bonding in female prairie voles (*Microtus ochrogaster*). Physiol. Behav. 80, 95–101. 10.1016/s0031-9384(03)00226-9 14568313

[B9] De Vries G. J. Panzica G. C. (2006). Sexual differentiation of central vasopressin and vasotocin systems in vertebrates: different. Neuroscience 138, 947–955. 10.1016/j.neuroscience.2005.07.050 16310321 PMC1457099

[B11] Dewan A. K. Maruska K. P. Tricas T. C. (2008). Arginine vasotocin neuronal phenotypes among congeneric territorial and shoaling reef butterflyfishes: species, sex and reproductive season comparisons. J. Neuroendocrinol. 20, 1382–1394. 10.1111/j.1365-2826.2008.01798.x 19094086

[B10] Dewan A. K. Tricas T. C. (2011). Arginine vasotocin neuronal phenotypes and their relationship to aggressive behavior in the territorial monogamous multiband butterflyfish, *Chaetodon multicinctus*. Brain Res. 1401, 74–84. 10.1016/j.brainres.2011.05.029 21676381

[B12] Faraway J. J. (2016). Extending the Linear Model with R: Generalized Linear, Mixed Effects and Nonparametric Regression Models. Boca Raton, FL: Chapman and Hall/CRC.

[B13] Fischer E. K. Roland A. B. Moskowitz N. A. Tapia E. E. Summers K. Coloma L. A. (2019). The neural basis of tadpole transport in poison frogs. bioRxiv [Preprint] 10.1101/630681 PMC6661358 31311480

[B14] Giora J. Malabarba L. R. (2009). *Brachyhypopomus gauderio*, new species, a new example of underestimated species diversity of electric fishes in the southern south america (Gymnotiformes: Hypopomidae). Zootaxa 2093, 60–68.

[B17] Goodson J. L. (2005). The vertebrate social behavior network: evolutionary themes and variations. Horm. Behav. 48, 11–22. 10.1016/j.yhbeh.2005.02.003 15885690 PMC2570781

[B16] Goodson J. L. Bass A. H. (2001). Social behavior functions and related anatomical characteristics of vasotocin/vasopressin systems in vertebrates. Brain Res. Rev. 35, 246–265. 10.1016/s0165-0173(01)00043-1 11423156

[B18] Goodson J. L. Evans A. K. (2004). Neural responses to territorial challenge and nonsocial stress in male song sparrows: segregation, integration, and modulation by a vasopressin V1 antagonist. Horm. Behav. 46, 371–381. 10.1016/j.yhbeh.2004.02.008 15465522

[B20] Goodson J. L. Evans A. K. Lindberg L. Allen C. D. (2005a). Neuro-evolutionary patterning of sociality. Proc. R. Soc. B Biol. Sci. 272, 227–235. 10.1098/rspb.2004.2892 15705546 PMC1634965

[B15] Goodson J. L. Evans A. K. Soma K. K. (2005b). Neural responses to aggressive challenge correlate with behavior in nonbreeding sparrows. Neuroreport 16, 1719–1723. 10.1097/01.wnr.0000183898.47160.15 16189485 PMC2596666

[B19] Goodson J. L. Kabelik D. (2009). Dynamic limbic networks and social diversity in vertebrates: from neural context to neuromodulatory patterning. Front. Neuroendocrinol. 30, 429–441. 10.1016/j.yfrne.2009.05.007 19520105 PMC2763925

[B21] Goodson J. L. Kelly A. M. Kingsbury M. A. (2012). Evolving nonapeptide mechanisms of gregariousness and social diversity in birds. Horm. Behav. 61, 239–250. 10.1016/j.yhbeh.2012.01.005 22269661 PMC3312996

[B22] Goodson J. L. Wang Y. (2006). Valence-sensitive neurons exhibit divergent functional profiles in gregarious and asocial species. Proc. Natl. Acad. Sci. U S A 103, 17013–17017. 10.1073/pnas.0606278103 17071744 PMC1636570

[B23] Greenwood A. K. Wark A. R. Fernald R. D. Hofmann H. A. (2008). Expression of arginine vasotocin in distinct preoptic regions is associated with dominant and subordinate behaviour in an African cichlid fish. Proc. R. Soc. B Biol. Sci. 275, 2393–2402. 10.1098/rspb.2008.0622 18628117 PMC2603226

[B24] Grober M. S. George A. A. Watkins K. K. Carneim L. A. Oliveira R. F. (2002). Forebrain AVT and courtship in a fish with male alternative reproductive tactics. Brain Res. Bull. 57, 423–425. 10.1016/s0361-9230(01)00704-3 11923002

[B25] Harrison X. A. Donaldson L. Correa-Cano M. E. Evans J. Fisher D. N. Goodwin C. E.. (2018). A brief introduction to mixed effects modelling and multi-model inference in ecology. PeerJ 6:e4794. 10.7717/peerj.4794 29844961 PMC5970551

[B26] Holmqvist B. I. Ekström P. (1995). Hypophysiotrophic systems in the brain of the Atlantic salmon. Neuronal innvervation of the pituitary and the origin of pituitary dopamine and nonapeptides identified by means of combined carbocyanine tract tracing and immunocytochemistry. J. Chem. Neuroanat. 8, 125–145. 10.1016/0891-0618(94)00041-q 7598813

[B27] Hopkins C. D. (1991). Hypopomus pinnicaudatus (Hypopomidae), a new species of gymnotiform fish from french guiana. Copeia 1991, 151–161. 10.2307/1446259

[B28] Johnson Z. V. Young L. J. (2017). Oxytocin and vasopressin neural networks: implications for social behavioral diversity and translational neuroscience. Neurosci. Biobehav. Rev. 76, 87–98. 10.1016/j.neubiorev.2017.01.034 28434591 PMC5407410

[B29] Kasper C. Colombo M. Aubin-Horth N. Taborsky B. (2018). Brain activation patterns following a cooperation opportunity in a highly social cichlid fish. Physiol. Behav. 195, 37–47. 10.1016/j.physbeh.2018.07.025 30056042

[B30] Kent M. Bell A. M. (2018). Changes in behavior and brain immediate early gene expression in male threespined sticklebacks as they become fathers. Horm. Behav. 97, 102–111. 10.1016/j.yhbeh.2017.11.002 29117505 PMC5771839

[B31] Kollack-Walker S. Newman S. W. (1995). Mating and agonistic behavior produce different patterns of FOS immunolabeling in the male syrian hamster brain. Neuroscience 66, 721–736. 10.1016/0306-4522(94)00563-k 7644033

[B32] Kovács K. J. (2008). Measurement of immediate-early gene activation- c-*fos* and beyond. J. Neuroendocrinol. 20, 665–672. 10.1111/j.1365-2826.2008.01734.x 18601687

[B33] Lin X. Itoga C. A. Taha S. Li M. H. Chen R. Sami K.. (2018). c-Fos mapping of brain regions activated by multi-modal and electric foot shock stress. Neurobiol. Stress 8, 92–102. 10.1016/j.ynstr.2018.02.001 29560385 PMC5857493

[B34] Loveland J. L. Fernald R. D. (2017). Differential activation of vasotocin neurons in contexts that elicit aggression and courtship. Behav. Brain Res. 317, 188–203. 10.1016/j.bbr.2016.09.008 27609648

[B35] Maler L. Sas E. Johnston S. Ellis W. (1991). An atlas of the brain of the electric fish Apteronotus leptorhynchus. J. Chem. Neuroanat. 4, 1–38. 10.1016/0891-0618(91)90030-g 2012682

[B36] Maruska K. P. Mizobe M. H. Tricas T. C. (2007). Sex and seasonal co-variation of arginine vasotocin (AVT) and gonadotropin-releasing hormone (GnRH) neurons in the brain of the halfspotted goby. Comp. Biochem. Physiol. Part A Mol. Integr. Physiol. 147, 129–144. 10.1016/j.cbpa.2006.12.019 17276115

[B37] Mendonça R. Soares M. C. Bshary R. Oliveira R. F. (2013). Arginine vasotocin neuronal phenotype and interspecific cooperative behaviour. Brain Behav. Evol. 82, 166–176. 10.1159/000354784 24107293

[B38] Migliaro A. Silva A. (2016). Melatonin regulates daily variations in electric behavior arousal in two species of weakly electric fish with different social structures. Brain Behav. Evol. 87, 232–241. 10.1159/000445494 27215902

[B39] Milo R. Phillips R. (2016). Cell Biology by the Numbers. New York, NY: Taylor and Francis Group.

[B40] Miranda M. Silva A. C. Stoddard P. K. (2008). Use of space as an indicator of social behavior and breeding systems in the gymnotiform electric fish brachyhypopomus pinnicaudatus. Environ. Biol. Fishes 83, 379–389. 10.1007/s10641-008-9358-2

[B41] Montag-Sallaz M. Welzl H. Kuhl D. Montag D. Schachner M. (1999). Novelty-induced increased expression of immediate-early genes c-*fos* and arg 3.1 in the mouse brain. J. Neurobiol. 38, 234–246. 10.1002/(sici)1097-4695(19990205)38:2<234::aid-neu6>3.3.co;2-7 10022569

[B42] Morton C. R. Hutchison W. D. (1989). Release of sensory neuropeptides in the spinal cord: studies with calcitonin gene-related peptide and galanin. Neuroscience 31, 807–815. 10.1016/0306-4522(89)90443-0 2480554

[B43] Nelson R. J. Trainor B. C. (2007). Neural mechanisms of aggression. Nat. Rev. Neurosci. 8, 536–546. 10.1038/nrn2174 17585306

[B44] Newman S. W. (1999). The medial extended amygdala in male reproductive behavior. A node in the mammalian social behavior network. Ann. N Y Acad. Sci. 877, 242–257. 10.1111/j.1749-6632.1999.tb09271.x 10415653

[B45] O’Connell L. A. Hofmann H. A. (2011). The vertebrate mesolimbic reward system and social behavior network: a comparative synthesis. J. Comp. Neurol. 519, 3599–3639. 10.1002/cne.22735 21800319

[B46] O’Connell L. A. Matthews B. J. Hofmann H. A. (2012). Isotocin regulates paternal care in a monogamous cichlid fish. Horm. Behav. 61, 725–733. 10.1016/j.yhbeh.2012.03.009 22498693

[B47] Okuyama T. Suehiro Y. Imada H. Shimada A. Naruse K. Takeda H.. (2011). Induction of c-fos transcription in the medaka brain (*Oryzias latipes*) in response to mating stimuli. Biochem. Biophys. Res. Commun. 404, 453–457. 10.1016/j.bbrc.2010.11.143 21138730

[B48] Ota Y. Ando H. Ueda H. Urano A. (1999). Differences in seasonal expression of neurohypophysial hormone genes in ordinary and precocious male masu salmon. Gen. Comp. Endocrinol. 116, 40–48. 10.1006/gcen.1999.7344 10525360

[B50] Perrone R. Batista G. Lorenzo D. Macadar O. Silva A. (2010). Vasotocin actions on electric behavior: interspecific, seasonal, and social context-dependent differences. Front. Behav. Neurosci. 4:52. 10.3389/fnbeh.2010.00052 20802858 PMC2928667

[B51] Perrone R. Macadar O. Silva A. (2009). Social electric signals in freely moving dyads of brachyhypopomus pinnicaudatus. J. Comp. Physiol. A Neuroethol. Sens. Neural Behav. Physiol. 195, 501–514. 10.1007/s00359-009-0427-6 19277680

[B49] Perrone R. Silva A. (2016). Vasotocin increases dominance in the weakly electric fish brachyhypopomus gauderio. J. Physiol. Paris 110, 119–126. 10.1016/j.jphysparis.2016.12.004 27940222

[B52] Pouso P. Radmilovich M. Silva A. (2017). An immunohistochemical study on the distribution of vasotocin neurons in the brain of two weakly electric fish, *Gymnotus omarorum* and *Brachyhypopomus gauderio*. Tissue Cell 49, 257–269. 10.1016/j.tice.2017.02.003 28242105

[B53] Ramallo M. R. Grober M. Cánepa M. M. Morandini L. Pandolfi M. (2012). Arginine-vasotocin expression and participation in reproduction and social behavior in males of the Cichlid fish *cichlasoma dimerus*. Gen. Comp. Endocrinol. 179, 221–231. 10.1016/j.ygcen.2012.08.015 22940647

[B54] Saito D. Komatsuda M. Urano A. (2004). Functional organization of preoptic vasotocin and isotocin neurons in the brain of rainbow trout: central and neurohypophysial projections of single neurons. Neuroscience 124, 973–984. 10.1016/j.neuroscience.2003.12.038 15026137

[B55] Schreibman M. P. Halpern L. R. (1980). The demonstration of neurophysin and arginine vasotocin by immunocytochemical methods in the brain and pituitary gland of the platyfish, *Xiphophorus maculatus*. Gen. Comp. Endocrinol. 40, 1–7. 10.1016/0016-6480(80)90089-1 7353780

[B56] Silva A. Quintana L. Galeano M. Errandonea P. (2003). Biogeography and breeding in gymnotiformes from uruguay. Environ. Biol. Fishes 66, 329–338. 10.1023/A:1023986600069

[B57] Silva A. Perrone R. Macadar O. (2007). Environmental, seasonal, and social modulations of basal activity in a weakly electric fish. Physiol. Behav. 90, 525–536. 10.1016/j.physbeh.2006.11.003 17178133

[B58] Stoddard P. K. (2002). The evolutionary origins of electric signal complexity. J. Physiol. Paris 96, 485–491. 10.1016/s0928-4257(03)00004-4 14692496

[B59] Teles M. C. Almeida O. Lopes J. S. Oliveira R. F. (2015). Social interactions elicit rapid shifts in functional connectivity in the social decision-making network of zebrafish. Proc. Biol. Sci. 282:20151099. 10.1098/rspb.2015.1099 26423839 PMC4614767

[B60] Thompson R. R. Walton J. C. (2004). Peptide effects on social behavior: effects of vasotocin and isotocin on social approach behavior in male goldfish (*Carassius auratus*). Behav. Neurosci. 118, 620–626. 10.1037/0735-7044.118.3.620 15174940

[B61] Wilson L. C. Goodson J. L. Kingsbury M. A. (2018). Neural responses to familiar conspecifics are modulated by a nonapeptide receptor in a winter flocking sparrow. Physiol. Behav. 196, 165–175. 10.1016/j.physbeh.2018.09.002 30196086

[B63] Wong C. J. H. (1997). Afferent and efferent connections of the diencephalic prepacemaker nucleus in the weakly electric fish, *Eigenmannia virescens*: interactions between the electromotor system and the neuroendocrine axis. J. Comp. Neurol. 383, 18–41. 10.1002/(sici)1096-9861(19970623)383:1<18::aid-cne2>3.0.co;2-o 9184983

[B62] Wong C. J. H. (2000). Electrical stimulation of the preoptic area in eigenmannia: evoked interruptions in the electric organ discharge. J. Comp. Physiol. A 186, 81–93. 10.1007/s003590050009 10659045

[B64] Zhong J. Liang M. Akther S. Higashida C. Tsuji T. Higashida H. (2014). c-*fos* expression in the paternal mouse brain induced by communicative interaction with maternal mates. Mol. Brain 7:66. 10.1186/s13041-014-0066-x 25208928 PMC4172782

[B65] Zubizarreta L. Perrone R. Stoddard P. K. Costa G. Silva A. C. (2012). Differential serotonergic modulation of two types of aggression in weakly electric fish. Front. Behav. Neurosci. 6:77. 10.3389/fnbeh.2012.00077 23181014 PMC3500767

