# Supplementary material for: Preoptic Area Activation and Vasotocin Involvement in the Reproductive Behavior of a Weakly Pulse-Type Electric Fish, Brachyhypopomus gauderio
Source: Front Integr Neurosci. 2019 Aug 13;13:37. doi: 10.3389/fnint.2019.00037 (PMC6700327; doi:10.3389/fnint.2019.00037)
Supplement: TABLE S1 — Detail of the main statistical analyses reported in the text. [file Data_Sheet_1.PDF]

Term	Chi squared	df	p-value
**#FOS+ cells**			
Isolated vs. social			
condition	0.17	1	0.681
POA section	1.52	1	0.218
condition X POA section	23.99	1	9.69e-07
**#AVT+ cells**			
Isolated vs. social			
condition	0	1	0.968
POA section	6.41	1	0.0113
condition X POA section	8.78	1	0.00305
#chirps			
#chirps	12.85	1	0.000338
POA section	77.28	1	1.49e-18
#chirps X POA section	17.72	1	2.56e-05
#movs. towards female			
#movs towards female	2.99	1	0.0838
POA section	64.47	1	9.79e-16
#movs towards female X POA section	9.51	1	0.00204
%time with female			
%time with female	4.61	1	0.0319
POA section	56.07	1	7e-14
%time with female X POA section	21.19	1	4.16e-06
**Proportion of FOS+/AVT+ cells**			
Isolated vs. social			
condition	1.93	1	0.165
POA section	0.14	1	0.713
condition X POA section	0.6	1	0.439
